# A sixth‐level habitat cascade increases biodiversity in an intertidal estuary

**DOI:** 10.1002/ece3.2499

**Published:** 2016-10-21

**Authors:** Mads S. Thomsen, Thomas Hildebrand, Paul M. South, Travis Foster, Alfonso Siciliano, Eliza Oldach, David R. Schiel

**Affiliations:** ^1^ Marine Ecology GroupSchool of Biological SciencesUniversity of CanterburyChristchurchNew Zealand; ^2^ School of Plant Biology & UWA Oceans InstituteUniversity of Western AustraliaCrawleyWAAustralia; ^3^ Cawthron InstituteNelsonNew Zealand; ^4^ Institute of Marine ScienceUniversity of AucklandAucklandNew Zealand

**Keywords:** epibiosis, facilitation cascade, indirect facilitation

## Abstract

Many studies have documented habitat cascades where two co‐occurring habitat‐forming species control biodiversity. However, more than two habitat‐formers could theoretically co‐occur. We here documented a sixth‐level habitat cascade from the Avon‐Heathcote Estuary, New Zealand, by correlating counts of attached inhabitants to the size and accumulated biomass of their biogenic hosts. These data revealed predictable sequences of habitat‐formation (=attachment space). First, the bivalve *Austrovenus* provided habitat for green seaweeds (*Ulva*) that provided habitat for trochid snails in a typical estuarine habitat cascade. However, the trochids also provided habitat for the nonnative bryozoan *Conopeum* that provided habitat for the red seaweed *Gigartina* that provided habitat for more trochids, thereby resetting the sequence of the habitat cascade, theoretically in perpetuity. *Austrovenus* is here the basal habitat‐former that controls this “long” cascade. The strength of facilitation increased with seaweed frond size, accumulated seaweed biomass, accumulated shell biomass but less with shell size. We also found that *Ulva* attached to all habitat‐formers, trochids attached to *Ulva* and *Gigartina,* and *Conopeum* and *Gigartina* predominately attached to trochids. These “affinities” for different habitat‐forming species probably reflect species‐specific traits of juveniles and adults. Finally, manipulative experiments confirmed that the amount of seaweed and trochids was important and consistent regulators of the habitat cascade in different estuarine environments. We also interpreted this cascade as a habitat‐formation network that describes the likelihood of an inhabitant being found attached to a specific habitat‐former. We conclude that the strength of the cascade increased with the amount of higher‐order habitat‐formers, with differences in form and function between higher and lower‐order habitat‐formers, and with the affinity of inhabitants for higher‐order habitat‐formers. We suggest that long habitat cascades are common where species traits allow for physical attachment to other species, such as in marine benthic systems and old forest.

## 1 Introduction

A habitat cascade is defined as an indirect positive effect on inhabitants (organisms found associated with habitat‐forming species) mediated by sequential formation or modification of biogenic habitat (Thomsen et al., 2010). For example, large first‐order habitat‐forming trees provide structural support to smaller second‐order habitat‐formers, such as orchids, mistletoes, nest epiphytes, and lichens, thereby indirectly facilitating bird and invertebrate inhabitants (Angelini & Silliman, 2014; Cruz‐Angon & Greenberg, 2005; Pettersson et al., 1995; Watson & Herring, 2012). Taxonomic studies of second‐order habitat‐formers have successfully predicted the existence of novel inhabitants (Darwin, 1862; Kritsky, 1991) and continue to reveal inhabitant species that are new to science (e.g., Henderson, Sultan, & Robertson, 2010; Rotheray, Hancock, & Marcos‐Garcia, 2007). More recently, field experiments have demonstrated community‐wide facilitation from second‐order habitat‐formers across a range of ecosystems and spatial scales (Altieri, Silliman, & Bertness, 2007; Angelini & Silliman, 2014; Bishop et al., 2012; Thomsen et al., 2010, 2016; Watson & Herring, 2012).

Yet, much less is known about habitat cascades than other types of indirect facilitation such as trophic cascades (Shurin, Gruner, & Hillebrand, 2006; Thomsen et al., 2010, 2016). Habitat cascades should occur where second‐order habitat‐formers are common and embedded within, entangled around, or attached to first‐order habitat‐formers, for example, along intertidal (Thomsen et al., 2016) and subtidal (Bell et al., 2014) rocky shores, in forests (Angelini & Silliman, 2014; Watson & Herring, 2012), seagrass beds (Edgar & Robertson, 1992; Gartner et al., 2013), mangroves (Bishop, Fraser, & Gribben, 2013; Bishop et al., 2012), salt marshes (Altieri et al., 2007; Angelini et al., 2015), and estuaries (Thomsen et al., 2010).

Determining the mechanisms that underpin interactions between habitat‐formers and their inhabitants is critical to understanding and predicting how such cascades vary across ecosystems, habitats, and environments. The strength of a habitat cascade should increase with the (1) Amount (abundance or size) of the second‐order habitat‐former, (2) Difference in form and function between the second and first‐order habitat‐formers, and (3) Affinity of the inhabitants for the second‐order habitat‐former, ranging from specialist (obligate) to generalist (facultative) affinities (Thomsen et al., 2010). These three factors (here referred to as the “ADA” model) are likely to operate simultaneously and should therefore be studied in concert. The *“*amount” part of the ADA‐model has been supported by manipulative and mensurative experiments (e.g., Angelini et al., 2015; Bishop et al., 2012; Thomsen, 2010). However, fewer studies have addressed whether form‐functional “differences” between habitat‐formers and “affinities” of inhabitants also regulate habitat cascades (but see Dijkstra, Boudreau, & Dionne, 2012; Hughes et al., 2014; Thomsen et al., 2013).

Furthermore, most studies to date have focused on size‐structured three‐level habitat cascades that include a large first‐order habitat‐former, a smaller second‐order habitat‐former, and a group of inhabitants. These studies test whether inhabitants are more commonly associated with coexisting first and second‐order habitat‐formers compared to first‐order habitat‐formers on their own (Altieri et al., 2007; Angelini & Silliman, 2014; Bishop et al., 2012; Thomsen et al., 2016). However, just like “long” consumption cascades in food web studies (Tronstad et al., 2010), habitat cascades may include more than three levels (Wahl, 1989). For example, there can be several nested levels of epiphytes in estuarine and rocky coastal ecosystems (Thomsen & McGlathery, 2005; Thomsen et al., 2016). Furthermore, not all habitat cascades are hierarchically size‐structured, because higher‐level habitat‐formers can be larger than the lower‐level habitat‐formers. For example, medium‐sized third‐order habitat‐forming genera, such as the seaweeds and the bryozoan *Bugula*, are often attached to the large second‐order habitat‐forming seaweed *Gracilaria vermiculophylla*, which itself is incorporated into the tubes of the small, first‐order habitat‐forming polychaete *Diopatra cuprea* (Thomsen & McGlathery, 2005). Although long habitat cascades are probably common, we are not aware of studies that have quantified them with rigorous sampling schemes.

Here, we address these research gaps by quantifying a long habitat cascade composed of bivalves, snails, bryozoans, and seaweeds, testing whether this cascade is regulated by the amount of habitat‐formers, and by measuring relative affinities of inhabitants for form‐functionally different co‐occurring habitat‐formers. We hypothesize that (1) the inhabitants are more abundant where there is more of each individual habitat‐former, (2) different inhabitants have different habitat affinities and thereby regulate habitat cascades differently, and (3) that these results are consistent across local environmental conditions, sites, and habitats.

## 2 Methods

### 2.1 Study system

The Avon‐Heathcote Estuary, located in Christchurch, New Zealand, is a ca. 8‐km^2^ shallow (average depth is 1.4 m) well‐mixed, nutrient‐rich estuary. The tidal regime is semidiurnal and ranges from 1.7 to 2.2 m. Salinity typically ranges from ca. 10 psu at the river mouths to 34 psu at the ocean during high tide. Seawater temperature varies annually from ca. 5°C in winter to 20°C in summer. We focused our research on five “model habitat‐formers”: the suspension feeding little neck clam *Austrovenus stutchburyi* (hereafter *Austrovenus*), the green ephemeral seaweeds *Ulva* spp. (hereafter *Ulva*)*,* mobile herbivorous trochid gastropods (hereafter “trochids”; *Micrelenchus tenebrosus* and *Diloma subrostrata*) (Jones & Marsden, 2005), the colonial and encrusting filter feeding nonnative bryozoan *Conopeum seurati* (hereafter *Conopeum*) (Inglis et al., 2006)*,* and the foliose perennial red seaweed *Gigartina atropurpurea* (hereafter *Gigartina*) (Nelson et al., 2014). These taxa represent a variety of habitat‐forming traits (e.g., common and rare, small and large, sessile and mobile, and with different modes of resource acquisitions), and all, except *Austrovenus*, have morphological structures that allow them to attach to hard surfaces and therefore also be “model inhabitants.” Mobile trochids were classified as “attached” inhabitants if they remained attached to their substrate if lightly disturbed (and trochid attachment can be stronger than the attachment of *Ulva*, Smith, 2006; Thomsen, 2004).

### 2.2 Surveys

We quantified the distribution of the four inhabitants associated with each of the five habitat‐formers in separate surveys (see Table 1 for an overview over sample sizes, sites, and collection dates; note that the first‐order habitat‐former *Austrovenus* is not an inhabitant because it does not have an attachment structure). Habitat‐formers were collected haphazardly and (except for trochids) bagged individually in the field. The inhabitants were counted under a dissecting microscope (×40 magnification; colonies, not individuals, of *Conopeum*) in the laboratory, and the biomass of each habitat‐former (except for trochids) was measured after drying to a constant weight at 55°C. We measured the shell length of all trochids and the shell dry weight on a subset of these snails, so we could convert all length data to biomass (gDW = 0.0004× (mm length^2.8917^); *R* ^2^ = .976, *n* = 200). *Conopeum* colonies were collected attached to trochids and scraped off with a scalpel before measuring its biomass. Finally, we collected another 30 small *Austrovenus* recruits (<0.6 cm width) from each of two sites, because this size class was absent in the first *Austrovenus* survey. We did not measure the specific width or biomass of these 60 small shells, and they were therefore excluded from correlation and regression analyses.

**Table 1:** Overview of intertidal survey data from the Avon‐Heathcote Estuary to identify potential linkages among five habitat‐formers and four inhabitants. Linear regressions were quantified between habitat‐formers and inhabitants from five surveys (see Figure 1a,c,e,g,i for corresponding scatter plots). Habitat‐formers and their inhabitants were collected from many sites, so they represent a broad suit of environmental conditions (* = widely scattered individuals were collected along 1.5‐km coastline). Survey 1 was performed from January to March 2013, survey 2, 4, and 5 from February to March 2016, and survey 3 from December 2014 to February 2015. Individually collected and bagged shells, seaweed fronds, and bryozoan colonies (*N*) were considered independent replicates. Acc dry weight (DW) and Acc counts = accumulated biomass and accumulated counts of all inhabitants. Linear regression models with high explanatory power (*r*_Pearson_ > .7) are in bold

Survey	Sites	Habitat‐former	vs.	Attached inhabitant	Habitat‐former total *N*	Habitat‐former Acc gDW	Inhabitant Acc counts	Total affinity (Acc counts/Acc gDW)	*r*_Pearson_	*p*_Pearson_	Linear slope
1	15	*Austrovenus*	vs.	*Ulva*	3,665	27,065	10,417	0.385	.201	<.001	0.164
		*Austrovenus*	vs.	Trochids	3,665	27,065	403	0.015	.150	<.001	0.011
2	10	***Ulva***	vs.	**Trochids**	**116**	90.6	**1,657**	**18.289**	**.796**	**<.001**	**17.780**
3	23	Trochids	vs.	*Ulva*	2,738	438.8	7,063	16.096	.268	<.001	8.903
		Trochids	vs.	*Conopeum*	2,738	438.8	68	0.155	.069	<.001	0.044
		Trochids	vs.	*Gigartina*	2,738	438.8	43	0.098	.041	.032	0.028
4	*	***Conopeum***	vs.	***Ulva***	**71**	**1.649**	**311**	**188.599**	**.707**	**<.001**	**186.036**
		*Conopeum*	vs.	Trochids	71	1.649	1	1.649	na	na	na
		*Conopeum*	vs.	*Gigartina*	71	1.649	39	23.651	.273	<.020	16.019
5	*	***Gigartina***	vs.	**Trochids**	**66**	**139.4**	**568**	**4.075**	**.741**	**<.001**	**2.732**
		*Gigartina*	vs.	*Conopeum*	66	139.4	1	0.007	na	na	na

### 2.3 Experiments

Three factorial experiments were performed in the intertidal zone in 0.25 × 0.25 m plots to identify potential mechanistic linkages among the habitat‐formers and their inhabitants. The experiments were repeated at different tidal elevations, locations, habitats or at different temporal scales to test whether habitat cascades are site‐ and habitat‐specific (see Table 2 for an overview over experimental designs, including experimental durations and sampling dates). Experimental plots were separated by at least 1 m, and all treatments were maintained every 10–14 days. When an experiment was terminated, a cylindrical core (10 cm inner diameter; 10 cm depth) was collected from the center of each plot. From each core, we counted trochids and *Ulva*,* Conopeum,* and *Gigartina* that were attached to the trochids and visible to the naked eye (we looked for, but found no other biogenic hosts, for these three inhabitant taxa)3, 4, 5.

**Table 2:** Overview of intertidal experiments from the Avon‐Heathcote Estuary to identify mechanistic linkages among five habitat‐formers and four inhabitants. Plots (0.25 × 0.25 m) were separated by at least 1 m. The experiments were repeated at different tidal elevations, distances from oceanic inlet, habitats (inside or outside *Zostera* seagrass bed), or temporal durations, to test whether habitat cascades are site‐ or habitat‐specific. At the end of the experiment, a cylindrical core (10 cm inner diameter; 10 cm depth) was collected from the center of each plot. All trochids and the *Ulva*,* Conopeum,* and *Gigartina* attached to trochids were counted (See Tables 3, 4, 5 for statistical analysis and Figures 2, 3, 4 for graphical analysis). 2A = ± removal of *Austrovenus* from plots. 2U = ± addition of loose *Ulva* fronds to plots. 5 Seaweeds = addition of loose *Ulva* and *Gracilaria* fronds to plots in low and high densities (plus a control without added seaweeds)

Experimental design	Plots	Dates	Total Trochids	Total *Ulva*	Total *Conopeum*	Total *Gigartina*
E1: 2 *A *×* *2 *U *×* *2 *Zostera* × 2 Elevations × 3 Rep	48	26/1 to 24/2‐2012	725	117	11	4
E2: 2 *A *×* *2 *U *×* *2 Distances × 2 Durations × 4 Rep	64	4/12‐2012 to 15/1 and 15/2 2013	587	80	10	7
E3: 5 Seaweeds × 2 *Zostera* × 5 Rep	50	25/1 to 17/2‐2013	1,074	406	24	15

We first tested whether *Austrovenus* and *Ulva* have consistent effects in different habitats and elevations. Forty‐eight plots were established on a mudflat and in an adjacent seagrass bed (24 plots per habitat) at two tidal heights separated vertically by ~10 cm. *Austrovenus* was removed (A‐), and *Ulva* was added (U+, removing attached trochids prior to additions) to plots in an orthogonal design (*n* = 3). The density of *Austrovenus* in unmanipulated control plots was 275 m^−2^ ± 48 (*n* = 24, densities were similar in the *Zostera* bed and the adjacent mudflat). Ca. 150 g WWof *Ulva* was added to each U+ plot and pegged to the substratum with five u‐shaped pegs (Thomsen, 2010). Any existing *Austrovenus* were carefully removed from each A‐plot by hand. We also removed *Austrovenus* in a 5‐cm buffer zone to reduce lateral recolonization. Five pegs were added to U‐plots, and we mimicked a search for *Austrovenus* in A+ without removing any shells, as procedural controls for experimental disturbances.

Second, we tested whether *Austrovenus* and *Ulva* have consistent effects at different distances from the mouth of the estuary and with two different experimental durations (42 vs. 72 days, see Table 2 for details). Thirty‐two experimental plots were established on each of two mudflats that were 1 or 1.8 km from the mouth of the Avon‐Heathcote Estuary. *Austrovenus* and *Ulva* were manipulated and maintained as in the first experiment (*n* = 4 for each of two sampling times). The density of *Austrovenus* in unmanipulated control plots was 395 m^−2^ ± 56 (*n* = 32).

Third, we tested whether the effects of the second‐order habitat‐formers are density‐dependent and consistent between species by comparing effects of *Ulva* to those of the coarsely branched red alga *Gracilaria chilensis*. Thirty plots were established on a mudflat and in an adjacent seagrass bed (15 plots per habitat). In each habitat, *Ulva* and *Gracilaria* were added as in the previous experiments to five plots in low (40 g WW, U1, G1) and high (150 g WW, U2, G2) abundances and five plots were kept free of seaweeds (0).

### 2.4 Statistical analysis

For the survey data, we treated each individual habitat‐former as an independent replicate (spatial and temporal effects were addressed in the experiments) (Gribben et al., 2009; Martins et al., 2014; Thyrring, Thomsen, & Wernberg, 2013; Voultsiadou, Pyrounaki, & Chintiroglou, 2007). From the survey data, we first plotted the number of inhabitants versus the biomass of individual habitat‐formers (= “individual size‐based affinity graphs”) and then plotted the number of accumulated inhabitants versus the accumulated biomass of the habitat‐formers (= “accumulated affinity curves”). The individual affinity plots were analyzed with linear regressions, representing simple model fitting analyses. The cumulative affinity curves were only evaluated qualitatively. We also calculated “total affinities” between pairs of inhabitants and habitat‐formers, by dividing the total number of counted inhabitant with the total sampled biomass for each habitat‐former.

For the experimental data, we treated each collected core as an independent replicate. Experimental data were analyzed with factorial ANOVA. Data were transformed to meet assumption of normality (Shapiro–Wilk tests, *p* > .26 for all test factors and experiments) and variance homogeneity 3, 4, 5(see Tables 3, 4, 5 for transformations and results). SNK tests were used to separate different treatment effects for experiment 3. There were insufficient observations of *Conopeum* and *Gigartina* for factorial ANOVA (see Table 2), so these responses were instead evaluated with Mann–Whitney tests to examine effects of second‐order habitat‐forming seaweeds only (pooling across orthogonal test factors; seaweed additions were the most important test factor on abundances of trochids and *Ulva* attached to trochids, 3, 4, 5see Tables 3, 4, 5). All analyses were performed in Unistat 5.6.

**Table 3:** Experiment 1: Effects of removing *Austrovenus* (A = first‐order habitat‐former) and adding loose *Ulva* (U = second‐order habitat‐former) inside and outside a *Zostera* bed (Z) at two elevations (E) on trochids and *Ulva* inhabitants attached to trochids. See Figure 2 for graphical analysis. Data were log (*x* + 1) transformed

Source	*df*	Trochids			Attached *Ulva*		
		SS	*F*	*p*	SS	*F*	*p*
*Austrovenus* (A)	1	0.039	0.44	.511	0.095	1.28	.267
*Ulva* (U)	1	6.983	78.58	**.000**	2.225	30.00	**.000**
*Zostera* (Z)	1	4.649	52.32	**.000**	1.166	15.73	**.000**
Elevation (E)	1	0.050	0.56	.460	0.000	0.01	.944
U × Z	1	0.007	0.08	.776	0.004	0.05	.826
U × A	1	0.207	2.33	.137	0.051	0.69	.412
U × E	1	0.061	0.69	.412	0.091	1.22	.277
Z × A	1	0.234	2.63	.115	0.184	2.48	.125
Z × E	1	0.003	0.03	.854	0.027	0.37	.549
A × E	1	0.008	0.09	.762	0.000	0.00	.951
U × Z × A	1	0.052	0.59	.448	0.106	1.44	.240
U × Z × E	1	0.003	0.04	.847	0.062	0.83	.368
U × A × E	1	0.028	0.31	.580	0.027	0.36	.553
Z × A × E	1	0.277	3.12	.087	0.048	0.64	.428
U × Z × A × E	1	0.001	0.01	.939	0.001	0.02	.888
Error	32	2.844			2.373		

Significant values (*p* < .05) are shown in bold.

**Table 4:** Experiment 2: Effects of removing *Austrovenus* (A = first‐order habitat‐former) and adding loose *Ulva* (U = second‐order habitat‐former) on trochids and *Ulva* attached to the trochids. The experiment was performed at two distances (D) from the ocean and over two periods of time (T). See Figure 3 for graphical analysis. Data were log (*x* + 1) transformed

Source	*df*	Trochids			Attached *Ulva*		
		SS	*F*	*p*	SS	*F*	*p*
*Austrovenus* (A)	1	0.223	4.40	**.041**	0.010	0.44	.510
*Ulva* (U)	1	14.760	291.29	**.000**	3.259	150.14	**.000**
Distance (D)	1	0.082	1.61	.211	0.085	3.93	.053
Time (T)	1	0.117	2.31	.135	0.028	1.29	.263
U × A	1	0.067	1.32	.256	0.002	0.11	.740
U × D	1	0.067	1.32	.256	0.014	0.63	.430
U × T	1	0.091	1.81	.185	0.000	0.00	.997
A × D	1	0.004	0.09	.772	0.085	3.93	.053
A × T	1	0.081	1.60	.212	0.099	4.54	**.038**
S × T	1	0.022	0.43	.516	0.015	0.71	.404
U × A × D	1	0.000	0.00	.977	0.014	0.63	.430
U × A × T	1	0.098	1.94	.170	0.022	1.00	.322
U × S × T	1	0.040	0.78	.381	0.030	1.40	.243
A × S × T	1	0.248	4.90	**.032**	0.015	0.71	.404
U × A × D × T	1	0.029	0.57	.453	0.030	1.40	.243
Error	48	2.432			1.042		

Significant values (*p* < .05) are shown in bold.

**Table 5:** Experiment 3: Effects of adding loose seaweed (S = second‐order habitat‐formers), here *Ulva* and *Gracilaria,* in high and low densities, inside and outside a *Zostera* bed (Z) on trochids, and *Ulva* inhabitants attached to trochids. See Figure 4 for graphical analysis. Data were square root transformed

Source	*df*	Trochids			Attached *Ulva*		
		SS	*F*	*p*	SS	*F*	*p*
Seaweed (S)	4	**151.97**	**24.62**	**.000**	**89.41**	**23.99**	**.000**
*Zostera* (Z)	1	5.58	3.61	.064	0.73	0.79	.379
W × Z	4	8.51	1.38	.258	5.80	1.56	.204
Error	40	61.71			37.25		

Significant values (*p* < .05) are shown in bold.

## 3 Results

### 3.1 Survey

**Figure 1:**
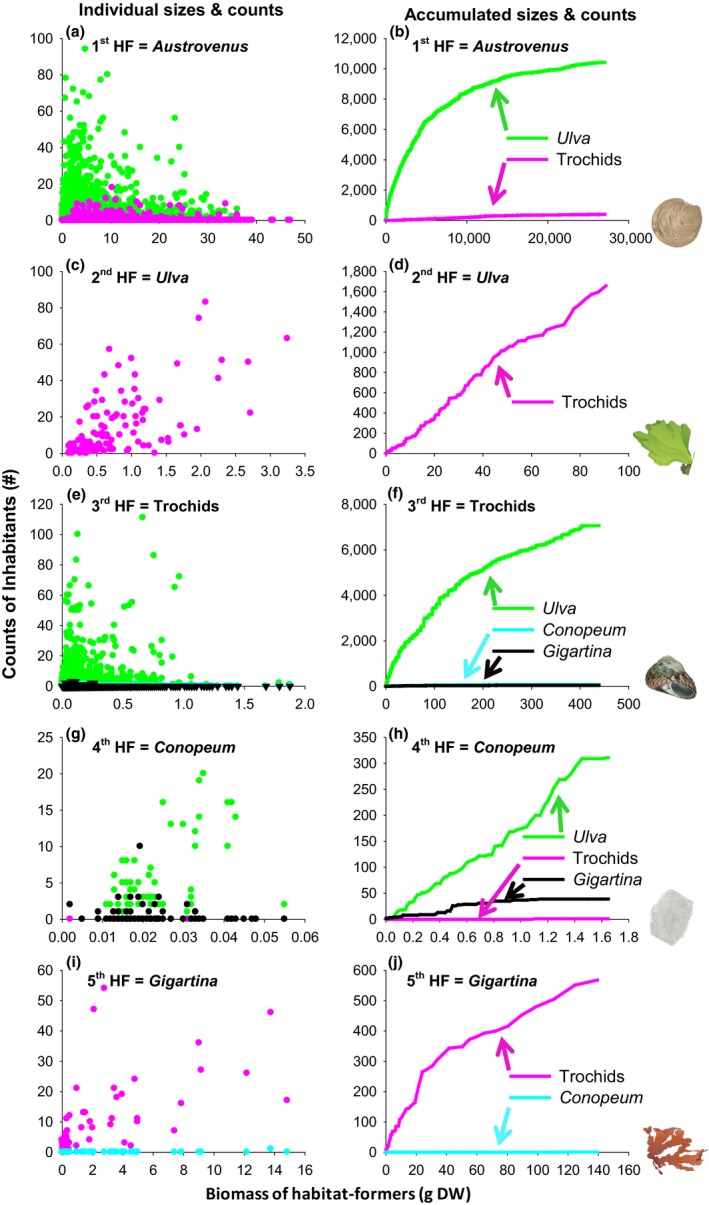
Abundances of four inhabitants (on y‐axis) attached to five habitat‐formers that co‐occur in the Avon‐Heathcote Estuary (HF, on x‐axis, including the bivalve *Austrovenus* (a‐b), seaweed *Ulva* (c‐d), trochid snails (e‐f), the bryozoan *Conopeum* (g‐h) and seaweed *Gigartina* (i‐j). Inhabitants were counted on individuals of each of the five habitat‐formers, before measuring the biomass of the habitat‐formers. Individual sizes and counts (a, c, e, g, i) = “individual affinity graphs” (see Table 1 for linear regressions and sample sizes); Accumulated sizes and counts (b, d, f, h, j) = “accumulated affinity curves” (derived from the individual affinity graphs)”

In the first survey, we counted >10,000 *Ulva* fronds and 403 trochids but no *Conopeum* or *Gigartina* attached to 3,665 *Austrovenus* shells (Table 1, Figure 1a,b; but we have observed a few *Gigartina* attached to *Austrovenus* over 4 years of frequent visits to the Avon‐Heathcote Estuary). We found a significant linear relationship between the size of *Austrovenus* and the abundance of *Ulva* and significant linear relationships between the size of *Austrovenus* and abundance of trochids (Table 1). However, *r* values were less than .21 implying poor fits (Figure 1a). The accumulated affinity curves (Figure 1b) highlighted that *Ulva* was orders of magnitude more common than trochids and that the slope of the *Ulva* curve decreased with increasing biomass (i.e., the number of inhabitants did not increase in proportion to the biomass increase of the host). We did not find a single attached inhabitant on any small (<0.6 cm) *Austrovenus* shells.

In the second survey, we counted >1,600 trochids attached to 116 *Ulva* fronds (Table 1, Figure 1c,d). We found a positive linear relationship between frond size and trochid abundance (Table 1, Figure 1c) and a near‐constant slope on the accumulated affinity curve (Figure 1d).

In the third survey, we counted >7,000 *Ulva* fronds, 68 *Conopeum* colonies, and 43 *Gigartina* fronds attached to 2,738 trochids (Table 1, Figure 1e,f). We found significant linear relationships between trochid shell size and the three inhabitants but with poor predictive fits (*r* values < .27, see Figure 3e). The slope of the accumulated affinity curve decreased with increasing accumulated biomass of the habitat‐former (Figure 1f).

In the fourth survey, we counted 311 *Ulva*, 1 trochid, and 39 *Gigartina* fronds attached to 71 *Conopeum* colonies. There were positive linear relationships between *Conopeum* colony size and *Ulva* (Table 1, Figure 1g), resulting in a near‐constant slope on accumulated biomass curve (Figure 1h). We also found a positive linear relationship between *Conopeum* colony size and abundances of *Gigartina* but with a poor fit (*r*_Pearson_ = .27, Table 1).

Finally, in the fifth survey, we counted 568 trochids and a single *Conopeum* colony attached to 66 *Gigartina* fronds (we have also observed *Ulva* to be attached to *Gigartina* during our frequent visits to the estuary). There was a positive linear relationship between *Gigartina* frond size and the number of trochids (Table 1, Figure 1i), and the accumulated affinity curve had initially a steep slope and then a more moderate slope (Figure 1d).

### 3.2 Experiments

**Figure 2:**
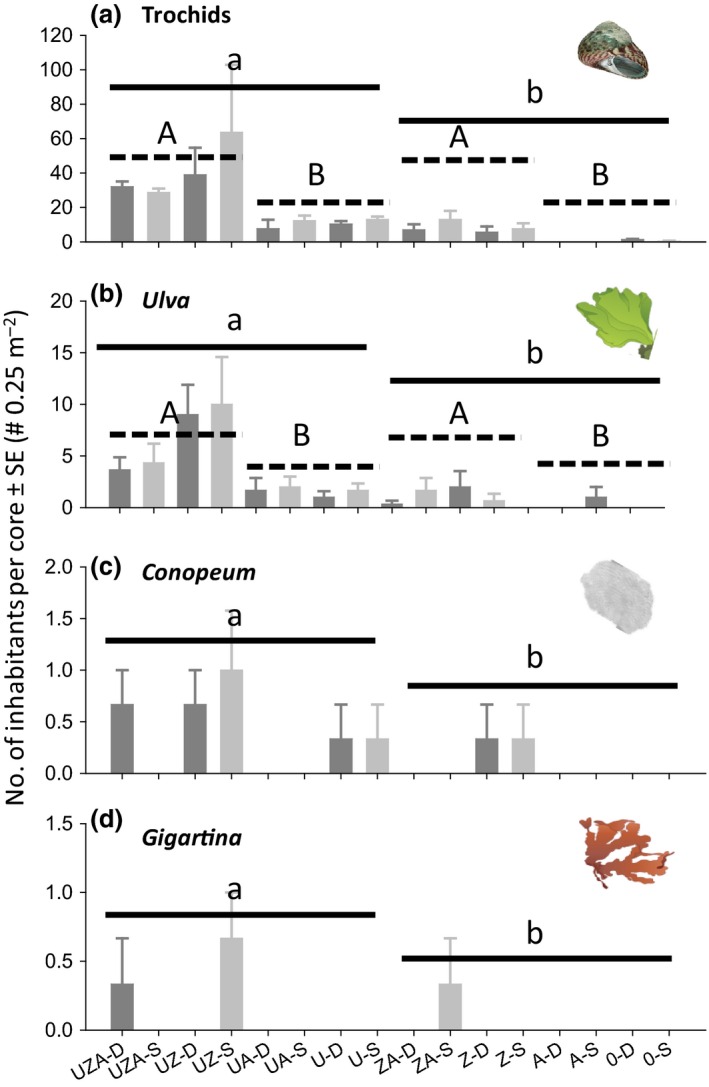
Experiment 1. Effects of removing *Austrovenus* (A on the *x*‐axis label = first‐order habitat‐former is present) and adding *Ulva* (U = second‐order habitat‐former is present), inside (Z) and outside a *Zostera* bed at deep (D; dark gray) and shallow (S; light gray) elevations on (a) trochids and inhabitants attached to trochids, including (b) *Ulva*, (c) *Conopeum,* and (d) *Gigartina*. See Tables 2 and 3 for experimental design and statistical analysis (*n* = 3, 0 on the *x*‐axis label = mud). Plots a‐b were analyzed with factorial ANOVA and c, d with Mann–Whitney tests on the “*Ulva*” test factor. Significant single factor effects (p < .05) of *Ulva* and *Zostera* are shown with solid and dashed lines, respectively

**Figure 3:**
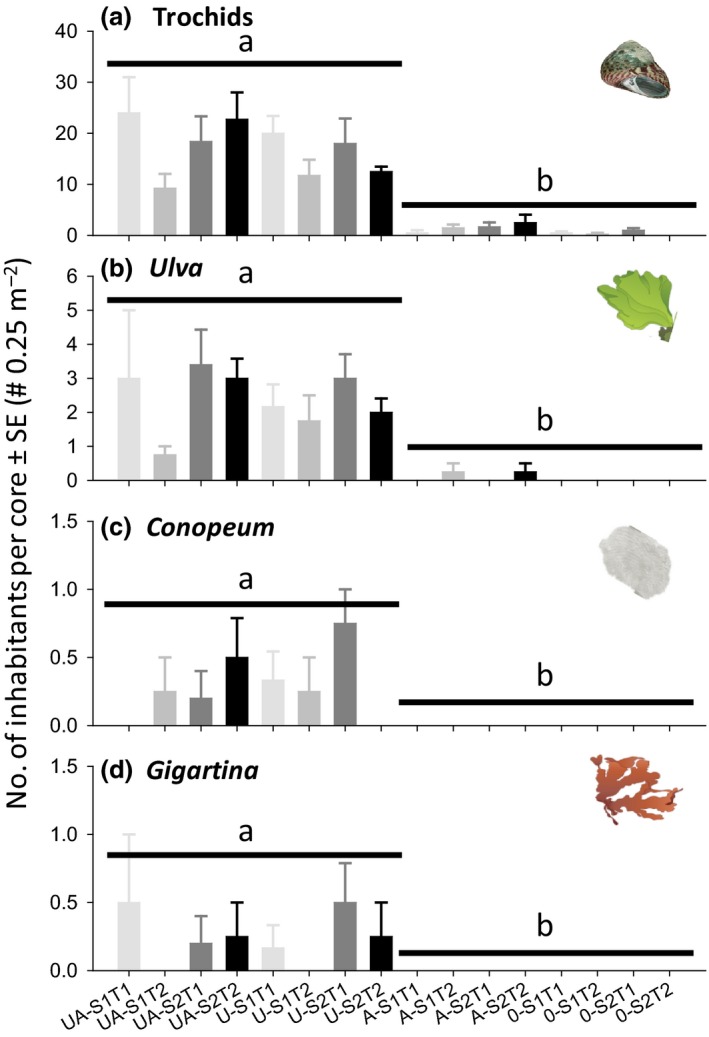
Experiment 2. Effects of removing *Austrovenus* (A on the *x*‐axis label = first‐order habitat‐former is present) and adding *Ulva* (U = second‐order habitat‐former is present) near (S1) and far (S2) from the ocean and after short (T1) and long (T2) exposure to habitat‐formers on (a) trochids and inhabitants attached to trochids, including (b) *Ulva*, (c) *Conopeum,* and (d) *Gigartina*. See Tables 2 and 4 for experimental design and statistical analysis (*n* = 4, 0 on the *x*‐axis label = mud). Plots a‐b were analyzed with factorial ANOVA and c‐d with Mann–Whitney tests on the “*Ulva*” test factor. Significant single factor effects of *Ulva* (p < .05) are shown with solid lines

**Figure 4:**
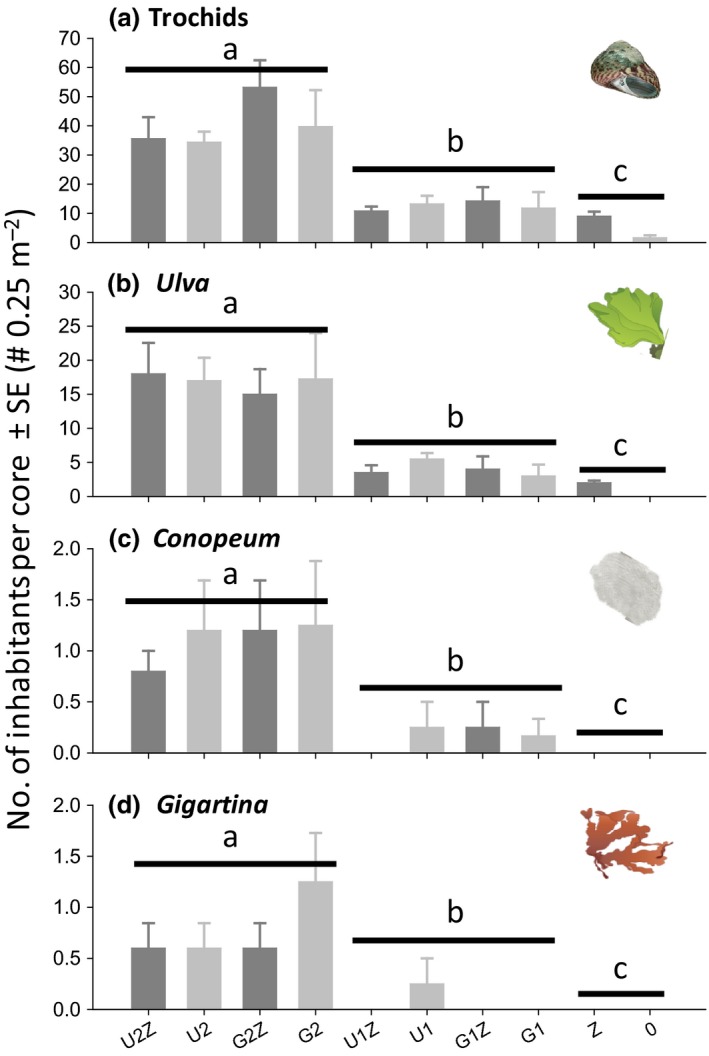
Experiment 3. Effects of adding *Ulva* and *Gracilaria* (U, G on the *x*‐axis label = second‐order habitat‐former is present) in high (2) and low (1) densities inside (Z) and outside a *Zostera* bed on (a) trochids and inhabitants attached to trochids, including (b) *Ulva*, (c) *Conopeum,* and (d) *Gigartina*. See Tables 2 and 5 for experimental design and statistical analysis (*n* = 5, 0 on the *x*‐axis label = mud). Plots a‐b were analyzed with factorial ANOVA and c‐d with Mann–Whitney tests on the “*Ulva*” test factor. Significant single factor effects of *Ulva* (p < .05) are shown with solid lines

We found, across the three experiments, consistent positive effects of second‐order habitat‐forming seaweeds on the abundances of trochids, *Ulva*,* Conopeum,* and *Gigartina* (Table 3, 4, 5, Figures 2, 3, 4). In all three experiments, the latter three inhabitants were only found attached to trochids.

More specifically, in experiment 1, we found higher abundances of trochids (Table 3, Figure 2a) in plots with (25.83 snails per core ± 5.83 *SE*) than without (4.38 ± 1.21) loose *Ulva*. There were also more trochids in the *Zostera* bed (24.58 ± 5.99) compared to the mudflat (5.63 ± 3.21). Similar patterns were found for *Ulva* attached to trochids (Figure 2b) with more attached *Ulva* in the presence of loose *Ulva* (4.17 fronds per core ± 0.97) compared to plots without *Ulva* (0.71 ± 0.27) and, again, in the *Zostera* bed (3.96 ± 0.20) compared to the mudflat (0.91 ± 0.05). We also found significantly more *Conopeum* colonies (Figure 2c) and *Gigartina* fronds (Figure 2d) in plots with than without *Ulva* (Mann–Whitney's *Z*‐test scores; *Conopeum Z* = −4.63, *p* < .001; *Gigartina Z* = 4.03, *p* < .001).

In experiment 2, we found again strong positive effects of adding loose *Ulva* to plots for both trochids (Table 4, Figure 3a; 16.87 snails per core ± 1.54 vs. 0.97 ± 0.26) and *Ulva* attached to the trochids (Figure 3b; 2.36 fronds per core ± 0.28 vs. 0.065 ± 0.05). We also found significant effects of removing *Austrovenus* and a complex 3‐way interaction between *Austrovenus* removals, distance, and experimental duration on the abundance of trochids (Table 4). However, these effects accounted for much less of the data variation compared to the manipulations of seaweeds (sum of squares: *Ulva* = 14.76 vs. *Austrovenus* = 0.22 and the 3‐way interaction = 0.03) and were therefore considered of little ecological relevance. We also found a significant 2‐way interaction between *Austrovenus* removals and experimental duration on the abundance of *Ulva* attached to trochids (Table 4). This effect, again, accounted for little variation compared to adding loose *Ulva* (sum of squares: *Ulva* = 3.26 vs. 2‐way interaction = 0.09). Again, we found significantly more *Conopeum* colonies (Figure 3c) and *Gigartina* fronds (Figure 3d) in plots with than without loose *Ulva* (Mann–Whitney's *Z*‐test scores; *Conopeum Z* = 6.80, *p* < .001; *Gigartina Z* = 6.47, *p* < .001).

In experiment 3, we found positive density‐dependent effects of adding both loose *Ulva* and *Gracilaria,* with similar effects between the two seaweed species, on abundances of both trochids and *Ulva* attached to trochids (Table 5). Thus, trochids and *Ulva* attached to trochids were most abundant in plots with high seaweed abundances, intermediate in low seaweed abundances, and lowest where there was no seaweed (Figure 4a; trochid snails per core = 40.79 ± 4.21 > 12.30 ± 1.88 > 5.00 ± 1.40; Figure 4b; *Ulva* fronds per core = 16.79 ± 2.08 > 3.85 ± 0.70 > 0.91 ± 0.34). Similar density dependency was found for *Conopeum* (Figure 4c) and *Gigartina* (Figure 4d) with highest abundances in the high seaweed densities and lowest abundances in control plots without seaweeds (Mann–Whitney's *Z*‐test scores; *Conopeum*:* Z*_zero‐low_ = −2.77, *p* = .004; *Z*_zero‐high_ = −4.44, *p* < .001, *Z*_low‐high_ = −4.72, *p* < .001; *Gigartina*:* Z*_zero‐low_ = −2.88, *p* = .0031; *Z*_zero‐high_ = −4.53, *p* < .001, *Z*_low‐high_ = −4.769, *p* < .001).

## 4 Discussion

**Figure 5:**
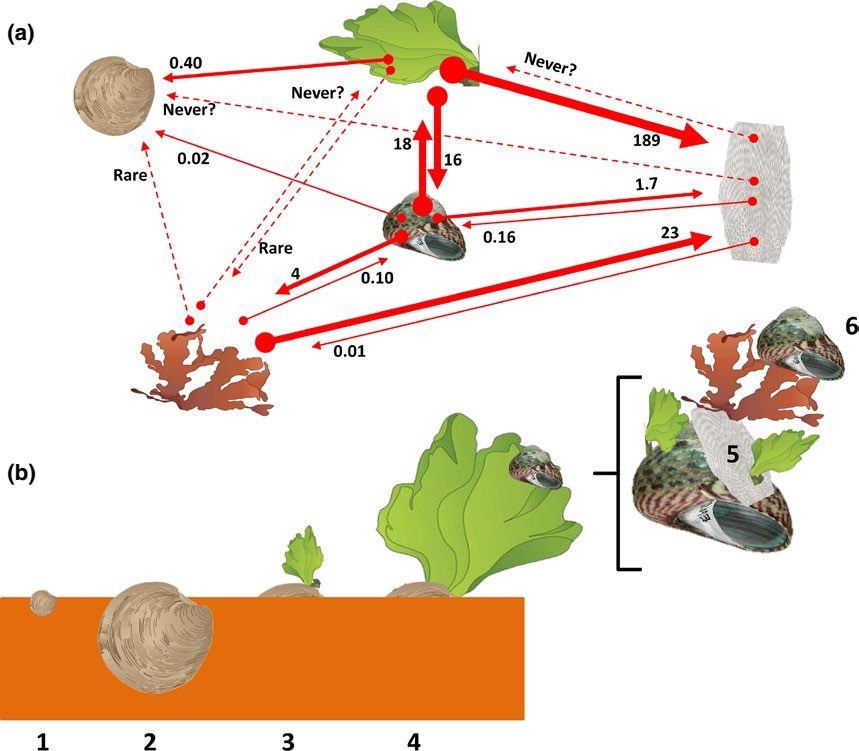
A sixth‐level long habitat cascade in the Avon‐Heathcote Estuary, New Zealand, portrayed as (a) a static habitat‐formation interaction network and (b) a succession of colonization events. a. The direction and thickness of arrows reflect the “total affinity” of inhabitants for habitat‐formers (the number next to the “attachment arrow head” = “Total Affinity” = number of attached inhabitants per gram dry weight habitat‐former, see Table 1 for calculations). “Rare” and “Never?” (dotted lines) represent plausible qualitative affinities that will require more sample intensive surveys to enumerate. *Austrovenus* cannot physically attach to organisms and has therefore no attachment arrows. b. 1. Juvenile *Austrovenus* colonize a mudflat, 2. *Austrovenus* grows into a large first‐order habitat‐former. 3. Shell protrudes above the sediment surface and *Ulva* attaches. 4. *Ulva* grows into a large second‐order habitat‐former that is colonized by trochids. 5. Close‐up of third‐order habitat‐forming trochid colonized by *Conopeum* (and *Ulva*), and 6. The fourth‐order habitat‐former *Conopeum* is colonized by the fifth‐order habitat‐forming *Gigartina* (and *Ulva*) which, again, in a loop, is colonized by trochids

We documented that (1) the amounts of higher‐order habitat‐formers, (2) form‐functional differences between habitat‐formers and inhabitant, and (3) inhabitants affinities for higher‐order habitat‐formers increased biodiversity in a sixth‐level long habitat cascade in the Avon‐Heathcote Estuary in New Zealand. This long habitat cascade can be interpreted as a static network with a ranked probability for facilitation at a point in time (Figure 5a) or as a temporal succession of events starting with the recruitment of the mollusc *Austrovenus,* the shells of which are colonized by other habitat‐formers over time (Figure 5b). *Austrovenus* is therefore, in this system, the essential basal habitat‐former (Hawes & Smith, 1995; Thomsen et al., 2010) that initiates this long habitat cascade. We also showed that this interaction network is regulated by the amounts of habitat‐formers and by form‐functional differences and affinities between habitat‐formers and inhabitants.

### 4.1 Amounts of habitat‐formers

Many ecological interactions are modified by the number of interactors present. For example, competition and trophic cascades typically depend on the densities of competitors and predators (Bellows, 1981; Schmitz, Hambäck, & Beckerman, 2000). We found analog effects for habitat cascades because the abundance of inhabitants increased dramatically when more biogenic habitat was sampled (Figure 1). This conclusion is supported by past field experiments that have documented a positive relationship between the abundance of second‐order habitat‐formers and their inhabitants (Figure 4, Angelini et al., 2015; Bishop et al., 2012; Thomsen, 2010). However, we only found strong size‐specific facilitation (positive slopes on the individual affinity curves, Figure 1) for the two seaweeds and the colonial bryozoan, but not the two shell‐forming molluscs. The latter result contrasts with other studies that have found strong size dependency of inhabitants attached to shells (Gribben et al., 2009; Martins et al., 2014; Thyrring et al., 2015; Wernberg et al., 2010). However, we often found other inhabitants on the shells such as anemones, barnacles, and limpets. It is possible that species interactions between these inhabitants, such as competition, or grazing, reduce size correlations. For example, limpets predominantly inhabit larger shells on which they likely exert considerable grazing pressure (Thomas et al., 1998; Wernberg et al., 2010). Furthermore, we did not find any inhabitants attached to very small *Austrovenus* recruits, highlighting that it takes time for inhabitants to colonize new biogenic substrates.

### 4.2 Form‐functional differences between habitat‐formers and inhabitant affinities

Different inhabitants had varied abundances on different co‐occurring habitat‐formers (Figures 1 and 5b). These differences are not only explained by the amount of available habitat (see above), but could also depend on the compatibility of traits between inhabitants and habitat‐formers. For example, trochids were abundant on seaweeds, probably because trochids are mobile grazers searching for food (alternatively, trochids may inhabit seaweeds to avoid predation and environmental stressors such as desiccation and temperature fluctuations). Sessile invertebrate inhabitants such as *Conopeum* can also select for certain substrates, but only at settlement (Walters & Wethey, 1991; Yoshioka, 1982). However, for the seaweeds, settlement is more likely a passive propagule rain (Santelices, 1990). Differences in affinities between inhabitants (Figure 5a) may reflect both presettlement processes such as early microbial inhibition (Dobretsov, Dahms, & Qian, 2006; Wahl, 1989), postsettlement processes such as competition for space and trophic interactions (Thomas et al., 1998) or physiological and biomechanical stress limitations (Thomsen, 2004; Thyrring et al., 2015). For example, *Ulva* is highly abundant in the Avon‐Heathcote Estuary (Hawes & Smith, 1995; Marsden & Bressington, 2009) and can produce a massive amount of propagules (Imchen, 2012; Yuanzi et al., 2014), and both juvenile and adults are resistant to estuarine stressors such as desiccation, low salinity, and partial burial (Liu et al., 2012; Vermaat & Sand‐Jensen, 1987). It is therefore not surprising that *Ulva* was the most abundant sessile inhabitant on all biogenic substrates. By contrast, *Conopeum* and *Gigartina* were only found attached to trochids. *Conopeum* and *Gigartina* are more typical of rocky shores than sedimentary euryhaline estuaries and, although we commonly found them attached to trochids, they are not included in the local comprehensive guide to species in the Avon‐Heathcote Estuary (Jones & Marsden, 2005). Our study of habitat cascades thereby demonstrated the existence of common, but inconspicuous, species (one of them being a nonnative species) in an otherwise well‐researched estuary. It is likely that traits of *Ulva* and *Austrovenus* make these two habitat‐formers poor substrates for *Gigartina* and *Conopeum*. For example, *Ulva* has rapid growth, a smooth surface, sheds epithallial cells and has an ephemeral life cycle (Geertz‐Hansen et al., 1993; Viaroli et al., 1996), whereas *Austrovenus* can actively migrate below the surface of the sediment in which it lives (Marsden, 2004). By contrast, trochids have hard surfaces and actively search for and inhabit *Ulva*, creating a microhabitat with relatively low sediment and desiccation stress. These traits allow sessile species such as *Gigartina* and *Conopeum* to attach to trochids and thereby coexist with other estuarine habitat‐formers.

### 4.3 Generality of the habitat cascade

We suggest that analogous habitat cascades are relatively common within and between estuaries. First, organisms were collected from many different locations in the Avon‐Heathcote Estuary, suggesting that different habitat‐formers commonly coexist in the same space. Second, experiments demonstrated that the second‐order habitat‐former *Ulva* could control the habitat cascade across a range of sites, times, and experimental durations. Third, we showed that a form‐functionally different seaweed species, *Gracilaria*, can provide similar habitat‐forming function (Davenport, Butler, & Cheshire, 1999; Littler, 1980; Tuya, Larsen, & Platt, 2011). The most important environmental modification was that the habitat cascade was stronger in seagrass beds compared to mudflats, probably because a higher baseline density of trochids in the seagrass bed (Figures 3 and 4) facilitated rapid movements from seagrass leaves onto *Ulva* fronds. Our results also support many other studies that show that estuarine seaweeds, throughout the world, facilitate epifaunal inhabitants (for meta‐analysis of how estuarine seaweeds facilitate epifauna, see Thomsen & Wernberg, 2015). We also suggest that analogous long habitat cascades are common in other estuaries, seagrass beds, and in rocky benthic systems. For example, in estuaries and other sedimentary habitats, first‐order mussels, cockles, oysters, and gardening polychaetes facilitate second‐order seaweeds, and barnacles that then facilitate third‐order habitat‐forming epiphytic seaweeds, tunicates, or sponges (Gribben et al., 2009; Thomsen & McGlathery, 2005; Yakovis, Artemieva, & Shunatova, 2008), and thereby likely support at least 4‐level habitat cascades. Similarly, second‐order habitat‐forming molluscs are common within seagrass beds (van der Heide et al., 2012) where they facilitate third‐order habitat‐forming bryozoan, sponges, barnacles, and seaweeds (Gribben et al., 2009; Thomsen et al., 2013). We have also recently observed long habitat cascades on rocky coastlines, where second‐order epiphytic seaweeds provide habitat to third‐order epiphytes (Thomsen et al., 2016) and turf‐forming algae facilitate kelps that provide habitat for shell‐forming gastropods that again provide structural support for epiphytic seaweeds and a variety of epifaunal animals (Thomsen, unpublished data).

### 4.4 Long size‐structured(?) habitat cascades in space and time

The “repeated habitat‐formation” documented in this long habitat cascade can be considered analogous to “repeated consumption” in long trophic cascades (Tronstad et al., 2010). Size‐structured biogenic habitat‐formation is well described from large to microscopic organisms. For example, large seaweeds (~1 m) can provide habitat for smaller tunicates (~0.1 m) (Wernberg et al., 2004) that can provide habitat for hydroids (~0.01 m) (Wernberg et al., 2004). Hydroids are also known to provide habitat for ciliates (~0.001 m) (Bavestrello et al., 2008) that can provide habitat for diatoms (~0.0001 m) (Totti et al., 2011) that, finally, can provide habitat for bacteria (~0.00001 m) (De Troch et al., 2012; Znachor, Šimek, & Nedoma, 2012). However, the habitat cascade documented here was not similarly size‐structured, because seaweeds often were much larger than the biogenic host they were attached to. Habitat cascades that are not size‐structured can occur where fluid forces are weak (as in estuaries) (Hawes & Smith, 1995; Thomsen, 2004). Conversely, in places dominated by strong wind or wave forces, large higher‐order habitat‐formers will increase drag, typically resulting in biomechanical failures of either the higher or lower‐order habitat‐formers, thereby reducing the probability of long term survival (Denny, 1999), and ultimately break down the habitat cascade.

We also found that some inhabitants attached to different habitat‐formers and at multiple levels in the habitat cascade, analogous to generalist and omnivorous consumers, respectively, in trophic cascades (Thompson et al., 2007; Williams & Martinez, 2004). For example, *Ulva* was found attached to all other habitat‐forming species at all levels in the habitat cascade (Figure 5a). *Ulva*'s broad habitat affinities make it difficult to summarize our results as a simple one‐dimensional interaction chain. The cascade studied here could alternatively be referred to as a habitat‐formation interaction web with feedbacks and loops (Figure 5a), just like interweaving trophic cascades are referred to as food webs (Bascompte & Melián, 2005). Alternatively, this “static” network interpretation could also be interpreted as a succession of events (Figure 5b) beginning with colonization by small *Austrovenus* that, over time, is colonized by *Ulva*, followed by trochids, *Conopeum* and *Gigartina*. Finally, we studied a habitat cascade mediated by physical attachment but higher‐order habitat‐formers can also be embedded within (Altieri et al., 2007; Angelini et al., 2015) or entangled around (Bishop et al., 2012; Thomsen, 2010) lower‐order habitat‐formers. The long habitat cascade in the Avon‐Heathcote Estuary could therefore be expanded to have yet another basal layer in places where *Zostera* provides habitat for *Austrovenus* (see experiments 1 and 3). Similar patterns of coexistence between seagrass and embedded shell‐forming molluscs have been reported from around the world (van der Heide et al., 2012).

### 4.5 Caveats and future studies

Despite our detailed surveys and experiments, the interaction web we quantified here is a gross simplification of how nontrophic habitat‐formation and modification regulate species distributions and community structures in the Avon‐Heathcote Estuary. First, we did not include microbes (e.g., bacteria, diatoms, protists) which can inhibit some and facilitate other links in the network (Dobretsov & Qian, 2006; Dobretsov et al., 2006; Wahl, 1989). Second, we have almost entirely ignored infaunal species (*Austrovenus* exempted). Third, the interaction network (Figure 5a) may obscure species‐specific effects (we pooled *Diloma* and *Micrelenchus* and tubular and sheet‐forming *Ulva*). Fourth, we ignored legacy effects from dead shells, common in the Avon‐Heathcote Estuary (Hawes & Smith, 1995) and in estuaries worldwide (Gutierrez et al., 2003). In contrast to habitat‐forming seaweeds that decompose rapidly (Duarte & Cebrián, 1986), calcareous shells can provide habitat for inhabitants for decades or centuries (Swinchatt, 1965). Fifth, we ignored complex interactions, such as when snails, in high densities, climb on top of each other (Wahl & Sonnichsen, 1992) or when human stressors modify species interactions (Smale & Wernberg, 2013). Finally, we only quantified links between five habitat‐forming taxa, thereby ignoring barnacles, limpets, hydroids, shell‐forming polychaetes, and at least six other seaweed species we have found attached to habitat‐formers in the Avon‐Heathcote Estuary. Addressing these caveats as well as experimentally quantifying species interactions, trait‐matching and feedbacks between habitat‐formers and inhabitants will provide a more realistic model over how habitat‐formation affects estuarine communities. Finally, we suggest that future studies should aim to determine the relative importance of, and test for interactions among, the components of the “ADA‐model”; that is, “amounts of higher‐order habitat‐formers,” “differences in form and function between lower and higher‐order habitat‐formers,” and “affinity of inhabitants for higher‐order habitat‐formers.” This simple model would become a much stronger predictive tool if co‐variation, interaction type (e.g., synergistic or additive), and variations across habitats and environmental conditions can be determined for the three attributes.

## 5 Conclusion

We documented a sixth‐level long habitat cascade where coexisting shell‐forming molluscs, seaweeds, and bryozoans were attached to each other in predictable sequences, thereby increasing biodiversity in our model system (compared to when habitat‐formers exist alone, Figure 5). We also found that the strength of facilitation, mediated through attachment space, increased with seaweed frond size, seaweed density, and shell density, but not shell size. This pattern was consistent across local environmental conditions, sites, and habitats. Inhabitants had varied affinities for different coexisting habitat‐formers, probably reflecting a combination of species‐specific traits associated with both juvenile and adult life stages, as well as the morphological and behavioral traits of the habitat‐formers. Long habitat cascades can thereby increase biodiversity on small scales compared to systems where organisms cannot attach to other organisms and could therefore be common in many marine benthic systems.

## Conflict of Interest

None declared.
